# A realist process evaluation of Enhanced Triple P for Baby and Mellow Bumps, within a Trial of Healthy Relationship Initiatives for the Very Early years (THRIVE): study protocol for a randomized controlled trial

**DOI:** 10.1186/s13063-019-3395-3

**Published:** 2019-06-13

**Authors:** Rosaleen O’Brien, Katie Buston, Daniel Wight, Elizabeth McGee, Jane White, Marion Henderson

**Affiliations:** 1 Glasgow Caledonian University, Psychology, Social Work and Allied Health Professionals, School of Health and Life Sciences, 4th Floor, George Moore Building, Cowcaddens Road, Glasgow, G4 0BA UK; 2 MRC/CSO Social and Public Health Sciences Unit, University of Glasgow, 200 Renfield Street, Glasgow, G2 3AX UK; 3 Public Health Sciences, NHS Health Scotland, Gyle Square, 1 Gyle Crescent, Edinburgh, EH12 9EB UK

**Keywords:** Process evaluation, Realist evaluation, RCT, Parenting interventions, Maternal mental health, Antenatal, Postnatal, Early years, Vulnerable populations

## Abstract

**Background:**

THRIVE is a three-arm randomised controlled trial (RCT) that aims to evaluate whether antenatal and early postnatal interventions, Enhanced Triple B for Baby (ETPB) plus care as usual (CAU) or Mellow Bumps (MB) plus CAU (versus CAU alone), can: 1) improve the mental health and well-being of pregnant women with complex health and social care needs; 2) improve mother-infant bonding and interaction; 3) reduce child maltreatment; and 4) improve child language acquisition. This paper focuses on THRIVE’s realist process evaluation, which is carefully monitoring what is happening in the RCT.

**Methods:**

Realistic evaluation provides the theoretical rationale for the process evaluation. We question: 1) how faithfully are MB and ETPB implemented? 2) What are the mechanisms by which they work, if they do, and who do they work for and how? 3) What contextual factors are necessary for the programmes to function, or might prevent them functioning?

The mixed-methods design includes quantitative measures, which are pre- and post-training/intervention questionnaires for facilitators and mothers-to-be, and post-session evaluation forms. Qualitative data collection methods include participant observation of facilitator training and the delivery of a series of antenatal sessions in selected intervention groups (*n =* 3 for ETPB and *n =* 3 for MB), semi-structured interviews with facilitators, pregnant women, partners, and referring facilitators, and telephone interviews examining the content of the postnatal components of ETPB and MB.

**Discussion:**

The findings of this process evaluation will help researchers and decision makers interpret the outcomes of THRIVE. It will provide a greater understanding of: how the interventions work (if they do); the extent and quality of their implementation; contextual factors facilitating and constraining intervention functioning; variations in response within and between subgroups of vulnerable parents; and benefits or unintended consequences of either intervention. Few studies to date have published detailed research protocols illustrating how realist process evaluation is designed and conducted as an integral part of a randomised controlled trial.

**Trial registration:**

ISRCTN, ISRCTN21656568. Registered on 8 November 2013.

**Electronic supplementary material:**

The online version of this article (10.1186/s13063-019-3395-3) contains supplementary material, which is available to authorized users.

## Background

This paper describes the planned process evaluation component of a Trial of Healthy Relationship Initiatives for the Very Early years (THRIVE), a three-arm randomised controlled trial (RCT) of two parenting interventions for vulnerable pregnant women. THRIVE is investigating whether receiving antenatal and early postnatal parenting interventions, in addition to care as usual (CAU), can improve maternal mental health, mother-infant relationships, and child language development compared with receiving routine antenatal care alone. The protocol for the trial and research questions for the outcomes evaluation can be found in the study documentation on the funder’s website [1]. The process evaluation will examine how faithfully Mellow Bumps (MB) and Enhanced Triple P for Baby (ETPB) are implemented, the mechanisms by which ETPB and MB work (if they work), who the interventions work for and how, and what contextual factors are necessary for the programmes to function as intended or prevent them functioning. The rigorous approach to evaluation outlined here is important and timely, given that it is current UK policy to invest significant resources in psychosocial parenting interventions in the antenatal and early postnatal period. The publication of protocols for process evaluations has particular utility for researchers who may be aware of recent guidelines for best practice [2] but lack examples of how to design and execute work of this complexity [3].

Women who are more vulnerable in pregnancy, due to domestic abuse, mental health problems, addictions, or a combination of complex social factors [4], are more likely to suffer from stress, depression, and/or anxiety, and produce higher levels of stress-related hormones [5]. Stress and mental health problems during pregnancy may disrupt a mother’s capacity to subsequently interact sensitively with her baby [6]. Poor quality interaction between mother and child, and maternal mental health problems, are also strong predictors of child maltreatment. Children who experience adversity (e.g. maltreatment) during the very early years demonstrate reduced language skills compared with their peers [7–16]. Other effects may last well into adulthood, reducing opportunities for educational attainment [17] and have negative impacts on emotional and mental well-being [18–21]. There is currently a paucity of evidence about whether and how parenting programmes targeting vulnerable families in the very early years are effective (or not) at improving maternal mental health and outcomes for children.

THRIVE is recruiting ‘vulnerable’ pregnant women (as defined by the Special Needs in Pregnancy protocol (SNiPS) of the Glasgow Child Protection Committee) at 20–30 weeks of pregnancy [22]. Participants will be referred through their antenatal booking clinics and randomly assigned to receive ETPB plus CAU, MB plus CAU, or CAU. The aims of the RCT are to evaluate whether ETPB plus CAU or MB plus CAU can: 1) improve maternal mental health and well-being; 2) improve mother-infant bonding and interaction; 3) reduce child maltreatment; and 4) improve child language acquisition. Children will also be followed up at 30 months to assess socio-emotional development and educational outcomes. The recent guidance of the Medical Research Council (MRC) highlights the importance of incorporating mixed-methods process evaluations into RCTs for the testing of complex interventions [2, 23]. The recommendation is that process evaluations include three key elements in their design: 1) data gathering to capture, and describe in detail, what happens during implementation; 2) identifying and investigating the mechanisms through which interventions operate; and 3) explaining the influence of ‘context’ (“which may include anything external to the intervention which impedes or strengthens its effects”) [2]. Reports of process evaluations have been criticised for not being explicit about theoretical influences and for lacking sufficient explanation of 1) what counts as context and 2) how intervention mechanisms and contextual elements interact [24, 25]. The broad principles of realist evaluation [26, 27] provides realist process evaluators with a framework that may make it easier to articulate, and test, the theoretical underpinnings of interventions like MB and ETPB (i.e. key components, intended mechanisms of action, and anticipated benefits) and make more explicit theorising about context-mechanism-outcome relationships (CMO configurations). Table 1 summarizes the approach of Pawson and Tilley [26] to realistic evaluation which has informed the design of THRIVE’s process evaluation.

**Table 1:** The key aims of realist evaluation

**Aim 1: Understanding the theory of programmes and how they work**	
1. Interventions are **theories** and will therefore have theories of change	
2. These theories are **embedded** in social systems	
3. They are **active** and effects are produced by, and require, the active engagement of actors	
4. They are also parts of **open systems**; there may be unanticipated events, political change, inter-programme and intra-programme interactions that influence how interventions bring about change	
**Aim 2: To understand and explain how, why, and for whom programmes work when implemented**	
5. Identify intervention **mechanisms**; emphasis on *how* an intervention works	
6. The importance of **context**; conditions in which the intervention is introduced and which are relevant to its functioning	
7. **Outcome patterns**; intended and unintended consequences	
8. **Context-mechanism-outcome pattern configurations (CMOs)** explaining the theory of how programmes activate mechanisms, amongst whom, and in what conditions. It is the **synergy** of CMO pattern configurations that is of interest, as opposed to merely separately identifying and describing ‘mechanisms’, ‘contexts’, and ‘outcomes’ Adapted from Pawson and Tilley [26]	

Although many researchers present them as diametrically opposed [28–30], experimental social science is viewed by some as being highly compatible with the methodological principles and epistemological assumptions of critical realism [31–33]. Recent discussions have highlighted the possibilities of designing ‘realist RCTs’ and suggest ways that realist principles might be integrated across all phases of the MRC framework [34]. Although there are excellent examples of how realist evaluation might be used for evaluation in different healthcare contexts (e.g. [35–39]), there have been very few protocols of realist process evaluations embedded within RCTs published to date.

THRIVE’s realist process evaluation aims to provide a greater understanding of: how the interventions work (if they do); the extent and quality of their implementation; contextual factors facilitating and constraining intervention functioning; variations in response within and between subgroups of vulnerable parents; and will explore benefits and/or unintended consequences of either intervention. In practice, it is a huge challenge, both methodologically and with the resources available, to design and execute a realist process evaluation that allows us to explore and document such complexities. We present a detailed protocol for THRIVE’s realist process evaluation, which explains how and why we designed this study the way we have, which may be of utility to other researchers designing realist process evaluations of RCTs.

## Methods

THRIVE is a 5-year RCT with a stratified randomised sample that ensures intervention groups will have a mix of identified vulnerabilities (see Additional files 1 and 2 for an overview of the main activities of the outcome and process evaluation). The trial and the mixed-methods realist process evaluation is led and managed at the MRC/Chief Scientist Office (CSO) Social and Public Health Sciences Unit, University of Glasgow, UK, following MRC guidelines. The lead researcher for the realist process evaluation is based at the School of Health and Life Sciences, Glasgow Caledonian University, UK. There is regular communication between the outcome and process evaluation teams, although we take advisement from a Data Monitoring Committee and a process evaluation advisory group about data management. Ethical approval was granted to conduct this research by the NHS’s West of Scotland Research Ethics Service, reference 13/WS/0163.

### Stage I of process evaluation design: understanding the theory of how ETPB and MB work

**Fig. 1:**
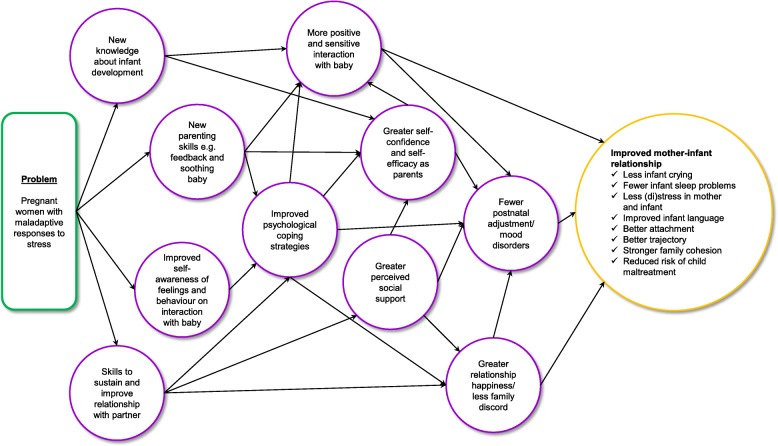
Enhanced Triple P for Baby: theory of change

**Fig. 2:**
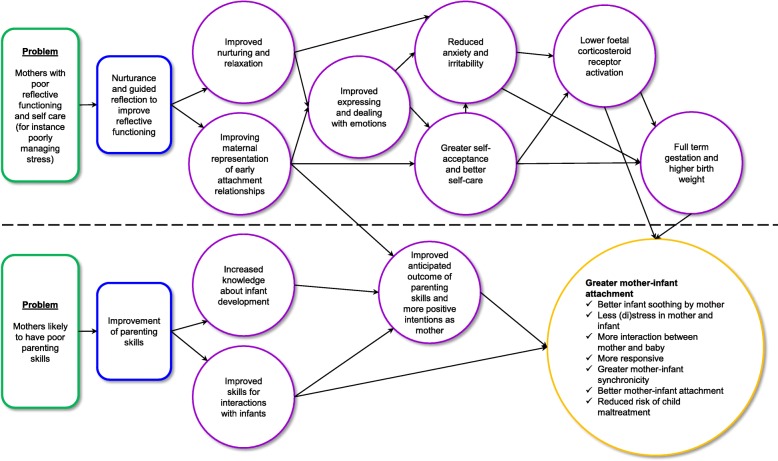
Mellow Bumps: theory of change

An important starting point for THRIVE’s realist process evaluation was to map the theory of change for each of the interventions (see Figs. 1 and 2 for ETPB and MB theory of change models), in other words to model how ETPB and MB are theorized to work and produce beneficial change (in line with Pawson and Tilley’s approach to realistic evaluation, as described under Aim 1 of Table 1). ETPB and MB were developed by researchers and facilitators with considerable expertise in designing parenting interventions. Both interventions are for women from 20 weeks of pregnancy and aim to reduce maladaptive responses to stress, maltreatment of children, and improve socio-developmental outcomes for children. However, there are fundamental differences in focus and mechanisms between ETPB and MB.

**Fig. 3:**
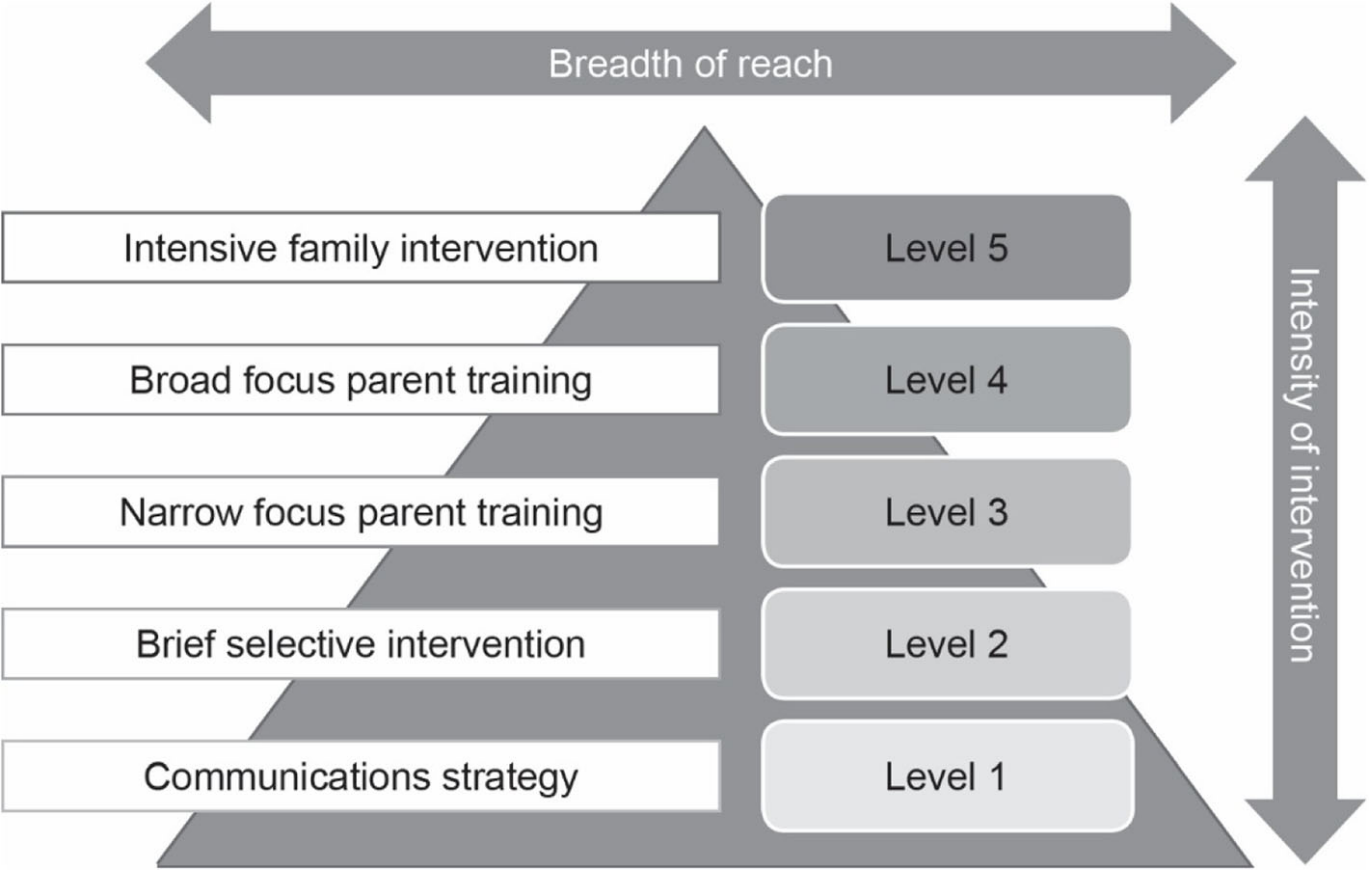
Levels of support provide by the Positive Parenting Programme. ©Triple P International (reproduced with written permission https://www.triplep.net/glo-en/find-out-about-triple-p/)

#### Developing theory of change models

In the developmental, planning, and organizational stages of THRIVE the team reviewed evidence on Triple P and Mellow Parenting interventions and intervention materials (where available). The Chief Investigator and Co-Investigators on THRIVE liaised with key stakeholders, including the developers of the interventions, to produce models of the theory of change underpinning ETPB and MB.

#### Enhanced Triple P for Baby (ETPB)

ETPB is the most recently developed variant of the University of Queensland’s Positive Parenting Program’s (Triple P) five-tiered standardized system of family support (see Fig. 3), which will be delivered and evaluated for the first time as part of THRIVE. ETPB, devised by Clinical Psychologists and underpinned by cognitive and behavioural theory, offers enhanced (Level 5) support to parents with ‘additional needs’.

The Triple P research group has published a wealth of studies and meta-analyses that report on the efficacy and effectiveness of other variants of Triple P (e.g. [40–46], see also: http://www.triplep.net/glo-en/home/). Triple P was appraised by NICE as being particularly effective for the treatment of conduct disorder based on five RCTs that delivered Standard (Level 3) and Self-directed and Enhanced levels of Triple P (Level 5), suggesting that Triple P may have particular benefits for families trying to address severe behavioural problems [47, 48].

Qualitative studies have largely been absent from evaluations of Triple P to date, which limits understanding of how and why these programmes work, for whom, and in what context, although there are a few very recent exceptions [49, 50]. Qualitative work has begun to make more explicit the behavioural changes that take place following participation in Triple P, for example praising children more often who, in turn, appeared more responsible and better at following instructions [49]. Qualitative research has also helped to identify necessary adaptations that are needed when transporting Triple P to a different social and cultural context [50].

Positive trial results have been questioned by researchers who were critical of ‘small sample sizes’ and who have suggested there is a ‘high risk of bias’ due to ‘lack of independence’ of Triple P evaluations, ‘poor reporting’, and ‘conflicts of interest’ [51–53]. However, Triple P evaluators argue that critical reviews (e.g. [51]) overlook the multiple levels, modes, and intensities of Triple P and fail to consider the broad range of possible outcomes generated by different variants of the programme [54]. A systematic review and meta-analysis of a wider sample of Triple P programmes found that there were short-term effects on a wide range of child, parent, and family outcomes, including children’s social, emotional, and behavioural outcomes, relationships, parenting satisfaction, and efficacy, as identified in parent observational data [55].

While still to be tested, ETPB aims to improve parents’ maladaptive psychological coping strategies for managing stress (see Fig. 1 for theory of change endorsed by Triple P International). Based on our observation of what was presented at facilitator training, and reading materials that were distributed about the programme, we know that parents will be offered four antenatal group sessions, each lasting 2 h, covering four ‘universal’ issues that commonly affect new parents. These include: 1) ‘positive parenting’; 2) ‘responding to your baby’ (education about infant development); 3) ‘survival skills’ (raising awareness of the connection between feelings and behaviour and how these impact on parents’ interactions with their baby); and 4) ‘partner support’ (development of skills to improve partner relationships and manage the stresses of parenting).

These sessions are theorized to improve psychological coping by offering new knowledge about infant development, new parenting skills (e.g. how to manage sleep, feeding, and soothing the baby), improved self-awareness of feelings and behaviour when interacting with the baby, and skills to improve and sustain the partner relationship. Postnatal support (up to three one-to-one sessions of 1 h and one group session) is introduced from 6 weeks after birth. Parents use this time to practice positive parenting skills and strategies with their baby in a naturalistic setting and facilitators provide them with feedback. Facilitators are trained to use a guided participation model, based on self-regulation theory, which encourages parents to identify their own solutions to problems. Therefore, self-management, self-efficacy, personal agency and problem solving appear to be implicit drivers of change. It is theorized that the outcomes of the ETPB include more positive and sensitive interaction with baby, greater self-confidence and efficacy as parents, fewer postnatal adjustment and mood disorders, greater perceived social support, and less discord in the partner relationship, which all result in a significantly improved mother-infant relationship.

A recent evaluation of another new Triple P programme, Baby Triple P, reports that mothers with postnatal depression (albeit a very small sample) found the delivery of an early postnatal parenting intervention highly acceptable [56]. The pilot RCT showed more favourable improvements in the intervention group on measures of depression, happiness, and perceptions of parenting a new baby, postpartum bonding, and parenting beliefs, although results were not significant. Another study of staff at a Mother and Baby Unit treating mothers presenting with severe mental illness also suggested that it would be feasible, and likely to be acceptable, to deliver Baby Triple P to women in this setting [57]. It remains to be seen how a wider ranging sample of vulnerable women respond to a similar parenting intervention and whether or not the enhanced component of Baby Triple P has any additive effects.

#### Mellow Bumps (MB)

MB, developed in Scotland, UK, is an antenatal intervention developed by Mellow Parenting (a group that is led by a research-active Clinical Psychologist). The social and cultural context of the area in which Mellow Parenting is based, an area of very high deprivation, was certainly influential to the initial development of support for women, although programmes are now delivered in a variety of settings and social contexts (as is the case for Triple P, which has international reach). MB was designed in response to the high levels of stress vulnerable women reported experiencing during and after pregnancy, as identified through early evaluation of Mellow Babies.

Current evidence on Mellow Parenting programmes includes a mix of peer-reviewed publications and grey literature [58–61] (also see http://www.mellowparenting.org/). Mellow Babies reportedly improved mother and child well-being and quality of the mother-child interaction, and was associated with an increased uptake of relevant services, including education, mental health, and social services [62]. Mothers who participated in Mellow Babies groups described a positive change in their perception of their children and an increased self-confidence in handling ‘difficult’ child behaviour [63]. Recent findings of a feasibility trial that compared Mellow Bumps, Chill out In Pregnancy (CHIP), and CAU suggest that MB may be effective at lowering maternal anxiety and mothers’ outwardly directed irritability [64]. However, there was a need for a definitive and well-powered [52] RCT to fully test these promising findings. The feasibility trial has been particularly informative about the procedures and processes needed to support THRIVE’s RCT.

MB offers six 2 h antenatal group sessions for pregnant women and an optional seventh session for fathers delivered in Week 5. The final group postnatal session is convened 6 weeks after birth. The principal aims of MB are to improve: 1) the reflective functioning and self-care (e.g. poorly managing stress) of pregnant women; and 2) parenting skills. MB is theorized to work by providing nurturance (e.g. improved nurturing and relaxation and support in expressing and dealing with emotions) and guided reflection to improve capacity to reflect on parenting (e.g. by improving maternal representations of early attachment relationships) [40]. Facilitators are trained to foster the nurturing and relaxing ethos of the group by conducting a ‘meet and greet’ session with participants in their homes prior to group attendance, inviting women to join a ‘closed’ and ‘safe’ group [58], and taking care of participants’ needs while at the group (e.g. welcoming them and offering refreshments on arrival). Facilitators devote time within each session to stress reduction (guided relaxation), which is intended to bring benefits to both mother-to-be and baby. Mothers-to-be are usually given a small gift during the session, for example bubble bath or massage oil to encourage self-care at home. The social aspect of the group (i.e. potentially gaining support from other mothers-to-be and non-judgemental facilitators) was found to be of therapeutic benefit in Mellow Babies groups [61], and may well have similar effects for participants of MB.

The theorized aims are to improve attachment and the quality of interaction between mother and baby; mothers-to-be are shown educational videos and play interactive games to learn about their baby’s capacity to respond to them whilst in the womb and during the very early postnatal period. Mothers-to-be are encouraged to interact with their bumps by being given torches to shine on their bumps to track movement of the baby and talking or singing to their bumps at home. The anticipated benefits of the intervention are reduced stress, anxiety, and irritability, greater self-acceptance, and better self-care and improved parenting skills and positive intentions, which reduce risks of child maltreatment and increase mother-infant attachment. See Fig. 2 for Mellow Bumps’ theory of change.

### Stage II of process evaluation design: key questions

We sought to address three overarching questions in Stage II of the realist process evaluation. These questions, outlined in Table 2, were designed to examine implementation fidelity and any contextual factors that influence delivery, as recommended by MRC guidelines on process evaluation [2]. We also questioned how theorized mechanisms of ETPB and MB were activated, amongst whom, and in what contexts, informed by the approach of Pawson and Tilley to realistic evaluation (see Aim 2, Table 1).

1. *How faithfully are MB and ETPB implemented?*

**Table 2:** Key questions for THRIVE’s realist process evaluation and how these will be examined

Key questions	Data source
**How faithfully are MB and ETPB implemented?**	
How well do facilitators feel they understand programme content and theory?	Observation of facilitator training and implementation, facilitator interviews, and facilitator self-reports on fidelity
How consistent are programme materials/protocols in directing facilitators what to deliver?	Observation of training and review of materials
What directions are facilitators given about adhering/adapting/personalizing the programme?	Observation of training and review of materials
How confident are facilitators about delivering ETPB or MB?	Observation of facilitator training and implementation, facilitator interviews, and facilitator self-reports on fidelity
What programme content is consistently covered? What content, if any, is missed out and why?	Facilitator self-reports on fidelity, supervisor interviews, and observation of selected groups during implementation
What work do facilitators have to undertake in order to deliver the intervention?	Interview at Time 2 (after facilitators gain experience of delivering groups)
How consistent are facilitators at delivering groups to completion?	Facilitator self-reports on fidelity, supervisor interviews, and observation of selected groups during implementation
What role does peer-assisted support (ETPB) and supervision (MB) have in fidelity?	Facilitator and supervisor interviews
To what extent do facilitator pairings (two deliver each group) affect delivery?	Facilitator self-reports on fidelity, supervisor interviews, and observation of selected groups during implementation
**What are the mechanisms by which MB and ETPB work, if they do, and who do they work for and how?**	
How does the programme’s theory of change explain the functioning of MB/ETPB?	Meetings with intervention developers and the research team to agree theory of change models
What information are facilitators given about programme theory and key mechanisms during training?	Observation of training, review of materials, facilitator interviews, and facilitator questionnaires
How do the programme materials/protocols explain programme theory and mechanisms?	Review of materials and observation of training
How well are key mechanisms understood by facilitators (at the point of training and later during implementation)?	Observation of facilitator training and selected groups during implementation, facilitator interviews, and facilitator self-reports on fidelity
What do facilitators think of the intervention and its key mechanisms?	Facilitator interviews, questionnaires, and facilitator post-session evaluation
How well do participants like the interventions?	Observation of selected groups, participant interviews, and questionnaires (facilitator’s perspectives will also be considered)
How do participants respond to knowledge on infant and child development in ETPB and MB?	Participant interviews and questionnaires, and observation of selected groups (facilitator’s perspectives will also be considered)
How do participants respond to parenting skills, partner skills content, and home-based practice in ETPB?	Participant interviews and questionnaires, and observation of selected groups (facilitator’s perspectives will also be considered)
How do participants respond to the nurturing aspects of MB, along with self-care, relaxation, and planned social activities in MB?	Participant interviews and questionnaires, and observation of selected groups (facilitator’s perspectives will also be considered)
How do participants respond to exploration of their past and present difficulties in MB?	Participant interviews and questionnaires, and observation of selected groups (facilitator’s perspectives will also be considered)
How does the experience of group sessions contribute to, or inhibit, the change mechanisms?	Participant interviews at Time 2 (3–12 months following birth)
**What contextual factors are necessary for the programmes to function, or might prevent them functioning?**	
What factors affect identification of suitable mothers-to-be and the referral process?	Interviews with referring practitioners, fieldworker observations, and interviews with managers
What are facilitator’s professional backgrounds?	Questionnaire and interviews with facilitators
What is the facilitators’ knowledge of, views on, and interest in MB/ETPB? Any experience of delivering parenting interventions?	Questionnaire and interviews with facilitators
What is the facilitators’ interest in, empathy and respect for, individual biographies and circumstances of mothers?	Interviews with facilitators and observation of training and selected groups during implementation
What are facilitators’ views on parenting interventions and what are their own experiences of parenting and of being parented?	Questionnaire and interviews with facilitators
How do facilitators manage MB/ETPB with other commitments?	Interviews with facilitators
How do facilitator managers view involvement with THRIVE?	Interviews with managers and facilitators
To what extent do facilitator pairings affect facilitators’ experiences of delivering ETPB and MB?	Interviews with facilitators and intervention developers/supervisors, post-session evaluation reports
How do facilitators feel about their engagement with, and response from, participants?	Interviews with facilitators and observation of selected groups during implementation
What is the suitability of venues for programme?	Observation of selected groups during implementation and post-session evaluation (facilitator and participant); interviews with participants and facilitators
What are mothers’ backgrounds (key relationships; social context; nature of social and health care needs)?	Mother-to-be questionnaires and interviews; observation of selected groups during implementation
How does mother engage with group?	Observation of selected groups during implementation and post-session evaluation (facilitator and participant); interviews with participants
What benefits does social interaction within the group offer participants? Are there any unintended consequences?	Observation of selected groups during implementation and post-session evaluation (facilitator and participant); interviews with participants
How do intervention group dynamics affect the experience of participation/retention?	Observation of selected groups during implementation and post-session evaluation (facilitator and participant); interviews with participants; interviews with non-attenders to explore reasons
How do vulnerabilities (e.g. mental health, drug use or stress) affect engagement?	Interviews with mothers-to-be and observation of groups; participant and facilitator post-session evaluation
How do material circumstances affect retention/adherence, e.g. poverty?	Interviews with mothers-to-be (attenders and non-attenders) and facilitator interviews Time 2
How do families affect adherence?	Interviews with mothers-to-be and observation of selected groups during implementation

*ETPB* Enhanced Triple B for Baby, *MB* Mellow Bumps

We question how faithfully ETPB and MB are implemented in relation to the developer manuals or protocols and will explore the wider contextual factors that may influence programme fidelity. Prior knowledge of, or experience of, delivering different levels or variants of Triple P programmes and/or Mellow Parenting programmes are two possible factors that could affect how faithfully interventions are delivered. Other influences may include the clarity and consistency of training and intervention materials and the amount of work that facilitators feel they need to undertake in order to familiarize themselves with content and deliver the interventions.

2. *What are the mechanisms by which* ETPB and MB *work, if they work, who do they work for and how?*

We seek to examine how specific mechanisms of ETPB and MB work for this particular population of pregnant women. We will compare the responses of participants and facilitators to the skills-based approach taken in ETPB with the nurturing and therapeutic approach taken by MB. We question the extent to which experiences within particular groups (e.g. in terms of vulnerabilities) result in the firing, or inhibition, of change mechanisms. We will also examine the decision making of referring facilitators about what to deliver, adapt, or leave out, and their sensitivity towards pregnant women.

3. *What contextual factors are necessary for the programmes to function, or might prevent them functioning?*

**Fig. 4:**
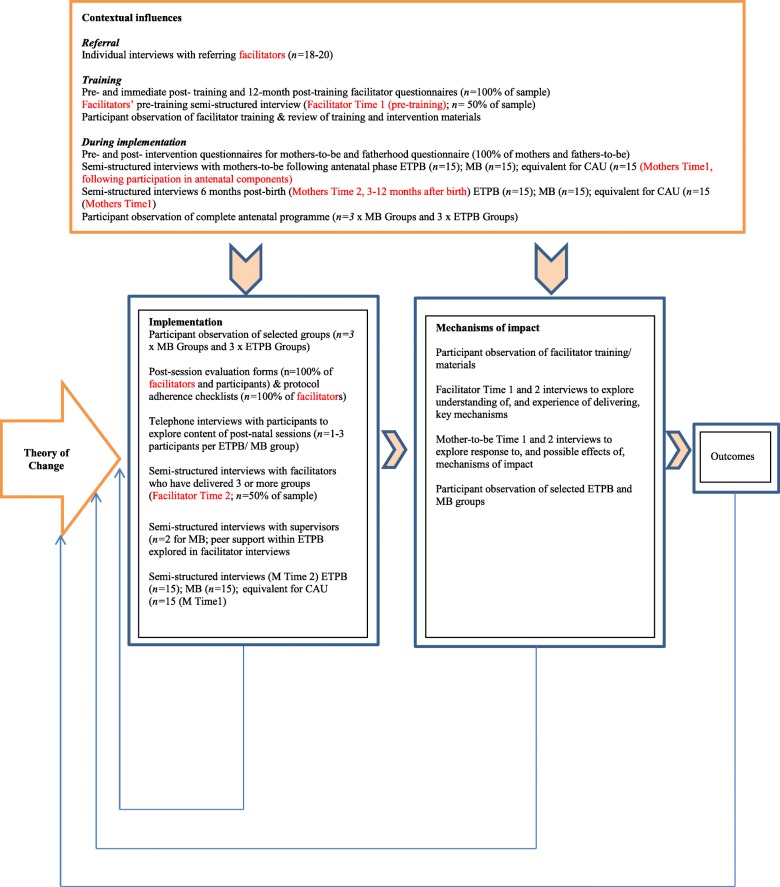
Main function and methods of the THRIVE realist process evaluation. Adapted from Moore et al. [23]. CAU care as usual, ETPB Enhanced Triple B for Baby, MB Mellow Bumps

It is important to explore the wider contextual factors that may influence the functioning of ETPB and MB, given that the effects of intervention come about through the interaction of their mechanisms with people and contexts [28]. We will examine the extent to which the professional backgrounds of facilitators, and prior knowledge of ETPB and/or MB, or previous training in these interventions, influence the way interventions are delivered and how they engage with participants. We also aim to locate the accounts of participants of their participation in ETPB or MB within a wider context of their lives and their experiences of parenting and of being parented. We question the extent to which group dynamics might be affected by the particular mix of vulnerabilities (e.g. homogenous or heterogeneous group composition). We aim to explore the nature of participants’ vulnerabilities in detail and question the extent to which ETPB, MB, or individual mechanisms within each are perceived to help. Figure 4 summarizes the main functions and methods of THRIVE’s realist process evaluation.

Multiple methods, including interviews, observations, and questionnaires, will be used to investigate the relationships between context, mechanism, and outcomes, thus allowing methodological triangulation [65].

We anticipate that the interview and ethnographic data collected as part of the realist process evaluation will be crucial in terms of understanding the complexities of the relationship between mechanisms, context, and outcomes, and will help explain how different characteristics of participants and facilitators, and different contexts, may mediate the intended benefits of MB and ETPB. Few qualitative studies have been published to date on Triple P or Mellow Parenting programmes that offer detailed insights into these critical processes. Ethnography in particular, it is argued, has an ‘untapped’ potential to yield new insights into the processes that influence the functioning of interventions [66], although it is still relatively rarely utilised. An ethnographic approach to process evaluation has recently been suggested as being particularly helpful for trials being conducted with vulnerable groups or challenging social contexts and where building theory might be important [67].

#### Recruitment to the RCT

Facilitators will be appointed by Greater Glasgow & Clyde (GGC) and Ayrshire & Arran (A&A) and allocated to ETPB or MB based on their stated preferences. It is planned that the facilitators will be line managed by their respective NHS Community Health Partnerships (GGC and A&A respectively). All facilitators in NHS A&A are likely to be recruited from Midwifery or Health Visiting background as this is the sustainable way NHS A&A envisage working with early parenting interventions in the future. NHS GGC have indicated they are facing too much demand for midwives’ time and wish to involve a wider range of professionals to undertake delivery of ETPB and MB. This contextual difference between the two NHS areas affords an opportunity to explore the impact of different strategic and managerial decision making on the rollout of the THRIVE interventions.

Mothers-to-be will be recruited from the same two NHS regions, enabling us to explore variations in the experiences of participants based on levels of deprivation and urban and rural localities. We plan to interview a sample of referring practitioners, to explore the referral process (e.g. how ‘vulnerability’ is conceptualized; how they identify mothers-to-be as suitable or ineligible for referral; what they think of ETPB and MB). Once a referral is made, mothers-to-be will be contacted by a member of the trial team who will obtain full consent to trial. This will cover consent for the process researcher to make initial contact with mothers-to-be, and partners, about interviews. Mothers-to-be are then randomized to MB plus CAU, ETPB plus CAU or CAU.

#### Data collection

##### Referring facilitators

We aim to conduct 18–20 semi-structured interviews with a sample of referring facilitators from clinic- and community-based settings. The sample design was responsive to any recruitment issues that came to the fore in the early stages of the trial, as reported by THRIVE field workers or the outcomes evaluation team in field notes. We purposively sampled the highest and lowest practitioners, from a range of clinic settings, to understand why recruitment was working well in some settings and not in others. We planned interviews with the Heads of Midwifery at Greater Glasgow and Clyde and Ayrshire and Arran to gain further insights into the ways in which the local context and organisational culture might have impacted recruitment.

##### Facilitators implementing ETPB or MB

- *Facilitator questionnaires*

Questionnaires (Facilitator Questionnaire 1) will be administered to all facilitators before they embark on training in MB or ETPB (to be delivered by Mellow Parenting and Triple P, respectively) to examine prior knowledge of, and views on, the intervention and their expectations of training. A second questionnaire (Facilitator Questionnaire 2) will be completed at the end of training. This examines views on course content, intervention materials, and perceived preparedness to deliver interventions groups.

(b). *Facilitator interviews*

Facilitators will be purposively sampled by area, intervention allocation, professional roles, and prior knowledge and experience of the interventions to be delivered. Pre-training interviews (Facilitator Time 1) will examine facilitators’ backgrounds in detail, motivations for applying for their post, and knowledge of, and thoughts on, the particular intervention they will be trained to deliver. The topic guide in Table 3 provides an initial framework for interviews, with flexibility to incorporate other relevant subject areas identified within and between interviews.

**Table 3:** Interviews with facilitators (Facilitator Time 1) examining key mechanisms and contextual factors

Tell me about your professional background and how your experience relates to current role	
What attracted you to this role?	
Previous experience of working with vulnerable families	
How do you envisage working with mothers-to-be and their partners likely to be recruited to THRIVE?	
Previous experience of delivering parenting groups	
What do you hope to get out of training?	
How do you feel about delivering MB or ETPB?	
Any prior knowledge of, or experience of delivering, MB or Triple P?	
Views of manager with regard to taking on this new role	

*ETPB* Enhanced Triple B for Baby, *MB* Mellow Bumps

Facilitators will be invited to participate in a second interview (Facilitator Time 2) once they have acquired experience of delivering the intervention (‘experienced’ meaning they have delivered three or more groups). Follow-up interviews will explore their experiences of delivering ETPB and MB and examine the influence of peer support (in the case of ETPB) or supervision (for MB). The topic guide in Table 4 will used as a starting point for these discussions.

**Table 4:** Interviews with facilitators (Facilitator Time 2) examining experiences of delivering ETPB/ MB

Describe experiences of delivering the intervention	
Explore understanding what the sessions consist of and how this developed	
Explore how closely the course content was adhered to	
Did you face any challenges when running the group sessions?	
Would you change anything about the interventions? If so, what and why?	
Were there any barriers to women/partners engaging in the sessions? If so, what?	
Who do they think group/individual sessions worked best for?	
Explore training and any refresher sessions. Did it equip you to deliver the groups? What work did you have to do following training to enable you to deliver the interventions?	
Experience of peer supervision (ETPB) or a supervisory process (MB)	

*ETPB* Enhanced Triple B for Baby, *MB* Mellow Bumps

Semi-structured interviews will be conducted after intervention delivery has begun to examine the experiences of MB developers in providing supervision.

(c). *Participant observation of facilitator training*

Participant observation will be conducted during ETPB (4 days) and MB (1 day) facilitator training, and implementation materials will be reviewed. Particular attention will be paid in training to the way key mechanisms are presented to, and understood by, facilitators. There may be opportunities during the course of training and other facilitator events (e.g. refresher training) to engage with facilitators informally (e.g. during lunch and coffee breaks) and discuss their initial thoughts about the programmes, check understandings of the theory of the interventions, and how they anticipate delivering groups. There may be further opportunities to gather ‘informal’ data (e.g. experiences of delivering groups) as the project team are likely to have ongoing contact with facilitators throughout the implementation period in order to arrange training updates and facilitate group organisation. Any relevant observations will be recorded in field notes.

Facilitators undertaking training in ETPB are given a detailed manual, with training and intervention materials and other materials (e.g. DVDs) that they need to deliver groups. The Triple P for Baby workbook, included with these materials, will be given to parents, along with copies of a number of shorter booklets (e.g. ‘Enhanced Triple P for Baby’ booklet). All materials will be shared with the research team following the delivery of facilitator training. Mellow Parenting will also provide facilitators and the research team access to all programme materials, which includes the MB manual and weekly packs prepared in advance to support the delivery of each session.

##### Pregnant women

- *Mothers-to-be questionnaires*

Pregnant women will be asked to complete a questionnaire at the first ‘meet and greet’ sessions (delivered as part of MB and ETPB) or at the beginning of the first group session (Mother’s Questionnaire, Time 1). The first questionnaire is designed to explore views on the intervention, thoughts on group participation, and anticipated outcomes of participation for themselves and their partners. A second questionnaire, administered to pregnant women at end of the last antenatal group session, examines the views of participants on the groups and what aspects of the intervention they think works (Mother’s Questionnaire Time 2). Facilitators will post questionnaires to participants who miss the relevant sessions. Participants who do not respond to postal questionnaires will be followed-up by telephone. Post-trial quantitative analyses will use the data on participant characteristics to investigate which participants benefitted most from the interventions.

b) *Mothers-to-be interviews*

Semi-structured individual interviews will be conducted at the end of the antenatal phase of ETPB and MB before the birth (Mothers Time 1). Interviews will be used to gather detailed background information about the pregnant women, including the nature of their additional health and social care needs, the circumstances surrounding their pregnancy, and their experiences of being a parent and of being parented. Other topics likely to be covered include experiences of recruitment to the trial, understanding of and responses to key mechanisms, and thoughts on, and experiences of, participating within the group (see Table 5 for topic guide).

**Table 5:** Interviews with mothers-to-be (Mothers Time 1) exploring key mechanisms and contextual factors

Relevant background information (e.g. relationship with partner; own parents; friends; other children) and circumstances surrounding the pregnancy	
Contact with services (care as usual) and support during pregnancy	
Understanding how they came to be referred to trial, the nature of additional needs, feelings about being referred	
Experience of attending sessions (e.g. atmosphere of groups; content, understanding of what key mechanisms were/what was supposed to be achieved)	
Relationships with participants and facilitators	
Has the nature of the group added to or taken away from content (dynamics; mix of vulnerabilities)?	
Partner/family response to participation	
To what extent has the intervention helped? In what ways? Any negative consequences?	
Hopes for motherhood	

Mothers will be re-contacted 6 months after the birth of their babies and invited to take part in a second individual interview (Mothers Time 2). Follow-up interviews will explore how their lives have been since their babies were born and whether or not there have been any sustained benefits of their involvement in THRIVE, or indeed any negative effects (see Table 6 for topic guide).

c) *Interviews with partners/fathers-to-be*

**Table 6:** Interviews with mothers-to-be (Mothers Time 2) exploring contextual factors that mediate outcomes

How has life been since last interview (and since baby was born)?	
Any changes/major life events since last interview	
Contact with services since birth (care as usual)	
Reflections on participating in the intervention	
Explore the legacy of the intervention	
Talk about any changes in the following as a result of the intervention (e.g. self-esteem; self-confidence generally and as a parent; anxiety generally and as a parent; self-accepting generally and as a parent; feelings of guilt generally and as a parent)	
Did the intervention improve/prompt change (e.g. knowledge/understanding of infant needs; attitudes regarding being a parent/child rearing (how); parenting skills; responding to baby’s needs; behaviour regarding partner (and his/her behaviour towards you); feelings; self-awareness; nature of social contact with group members; on-going or new support? Any feelings arising about own childhood and how parented?)	

MRC funding was secured for a complementary PhD studentship to study the experiences of marginalised fathers who will be sampled from the trial. Interviews will be conducted as part of the process evaluation to explore: 1) how partners might influence the participation of mothers-to-be in the trial; 2) the involvement of partners (fathers, same-sex parents, sisters, and mothers can accompany the participant to each session in ETPB); and 3) fathers’ engagement with MB, if any (there is an optional ‘Dad’s session’ for one of seven possible sessions). Table 7 summarises our procedures for collecting and analysing qualitative data.

**Table 7:** Treatment of qualitative data

Participants will be asked to give their written consent for interviews to be audio recorded	
Interviews will be conducted at a time convenient for participants, usually in their home	
Interviews will be transcribed verbatim, anonymised, and cross-checked for quality	
Field notes will be written immediately following interviews to note unrecorded discussion and reflect on key themes	
The main themes will be independently coded across a sample of interviews by two researchers using Nvivo 10	
A coding frame will be agreed by two researchers	
All data will be coded using this coding frame and further additions/adaptions will be discussed	
Themes will be summarized using the one sheet of paper method (OSOPs) [40] Analysis of themes will be developed through writing and wider discussion with the process team	

##### Implementation of ETPB and MB

- *Participant observation of MB and ETPB*

Participant observation was suggested to MB programme developers who found that other data collection methods (e.g. videoing group sessions or a researcher attending to observe) were too intrusive and not a good fit with the programme ethos. However, a researcher fully participating in sessions was considered more sensitive and was accepted by both MB and ETPB developers as a way to understand how the programmes work. The precise role the researcher takes within these groups is likely to be negotiated with the intervention developers, facilitators, and group participants. The process researcher will participate in three complete series of antenatal sessions with three different MB groups and three different ETPB groups. Groups will be selected at the early, mid-point, and end stages of the trial to ensure we capture facilitators with a range of different experiences of implementing groups (e.g. implementing their first group through to experienced facilitator). Table 8 indicates the subjects of interest that are most likely to be noted. Data will take the form of field notes, which will be written from memory immediately after each group session. Researchers will be reflexive in their written accounts about the roles and the influence their presence has within groups.

b) *Post-session evaluation questionnaires*

**Table 8:** Participant observation of selected ETPB and MB groups

Setting up	
Getting there (transport), reception on arrival, comfort of room and facilities	
What session is being delivered: content and materials, including how closely this complies with MB/ETPB packs/content of training	
Who is delivering session?	
How many mothers-to-be (and partners) in attendance?	
Style of facilitator	
How mothers-to-be/parents interact with each other	
Facilitator/mother-to-be/parent interaction	
Level and nature of participation	
Facilitator style	
Key mechanisms. How do participants react to these?	
Response to any home-based tasks that may have been set between sessions	
Atmosphere/dynamics of the group	
What happens when the class ends?	
Researcher’s role, as negotiated with facilitators and participants	

*ETPB* Enhanced Triple B for Baby, *MB* Mellow Bumps

Facilitators and participants will be asked to complete post-session evaluation questionnaires after each intervention session. These invite respondents to review the content covered in each session and to reflect on what they think worked well and what was less successful. Responses are likely to inform the facilitator and participant interviews (Facilitator Questionnaire Time 2 and Mothers Questionnaire Time 2).

c) *Telephone interviews*

ETPB offer up to three postnatal home visits to each parent, or couple, from 6 weeks following the birth of the baby, and a final group session. Facilitators and participants will be asked to complete post-session evaluation forms for these home-based sessions just as they do for the group sessions above. We will include an additional measure of a short telephone interview (10–15 min) to further explore, with selected participants, the content of home-based postnatal sessions. Up to three women from each ETPB group will be contacted and asked to describe one of three sessions. Data will take the form of research notes recorded on a pro forma observation sheet.

#### Analysis

All process data will be analysed independently of the outcome data and, importantly, documented before the outcomes are actually known [2]. Pawson and Tilley state that the aim of analysis of realist evaluation data is to ‘draw closer to explaining the complex signature of outcomes’ that an intervention may produce [27]. They suggest the starting point for analysis is to examine whether the theories about how each of the programmes work are supported or refuted by the data. They suggest that analysis is an ‘ever-repeating cycle’ of examining recognisable outcome variations, disentangling those that are less clear, and identifying unanticipated consequences of the intervention(s). This will involve constant comparison of emergent findings from the quantitative and qualitative studies outlined above.

Descriptive accounts of the data will be prepared in the order of completion of each key ‘project’ (e.g. pre-training interviews; observations; interviews with pregnant women) as far as is practicable. Report writing is likely to be a critical part of the process evaluation since it will enable us to: 1) keep clear records of which substantive themes were identified and when; 2) present what we thought about the data at particular stages of the evaluation (as opposed to re-interpreting data retrospectively in light of later analyses); and 3) compare data more easily. Dissemination activities (e.g. presentations and publication) will be carefully timed to avoid contamination of the RCT.

## Discussion

This trial is of great social importance since the overarching aim is to establish the most effective way to break the cycle of maladaptive coping leading to maltreatment of infants. The evaluation as a whole will make a substantial contribution to the evidence base for very early interventions appropriate to vulnerable pregnant women. The different theoretical perspectives of the interventions provide the opportunity to assess which theoretical approach has greatest empirical support. The mixed-methods realist process evaluation described here will help disentangle which components of each intervention work best, for whom, and why, and help explain the effects of antenatal and postnatal components of the intervention. The independence of the THRIVE team is important given that Triple P and Mellow Parenting’s involvement in the development and evaluation of their own programmes has attracted heavy criticism [49].

A trial of this scale and complexity inevitably presents practical and methodological challenges to both the trial and process teams. We have outlined here the work we hope to be able to do for THRIVE’s realist process evaluation. However, it will be important to remain flexible about the research design and be responsive to what happens within the RCT. It may be that emergent issues, or additional research problems, come to light during the course of implementation, meaning that planned methods have to be re-designed or that new measures have to be incorporated.

## Additional files

Additional file 1: SPIRIT checklist. Standard Protocol Items: Recommendations for Interventional Trials (SPIRIT) checklist indicating the location of relevant information with this publication. (DOC 123 kb)

Additional file 2: SPIRIT Schedule of activities. Schedule of activities for women participating in THRIVE and for MB and ETPB facilitators. Activities relating to the realist process evaluation of THRIVE are shown with bold text and shading. (DOCX 22 kb)
