# Supplementary material for: A realist process evaluation of Enhanced Triple P for Baby and Mellow Bumps, within a Trial of Healthy Relationship Initiatives for the Very Early years (THRIVE): study protocol for a randomized controlled trial
Source: Trials. 2019 Jun 13;20:351. doi: 10.1186/s13063-019-3395-3 (PMC6567913; doi:10.1186/s13063-019-3395-3)
Supplement: Supplementary file 2 — SPIRIT Schedule of activities. Schedule of activities for women participating in THRIVE and for MB and ETPB facilitators. Activities relating to the realist process evaluation of THRIVE are shown with bold text and shading. (DOCX 22 kb) [file 13063_2019_3395_MOESM2_ESM.docx]

**Schedule of activities for women participating in THRIVE and for MB and ETPB facilitators (outcomes and process evaluation)**

*Activities relating to the realist process evaluation of THRIVE are highlightedwith bold text and shading*

| **SCHEDULE OF ACTIVITIES FOR WOMEN PARTICIPATING IN THRIVE** | | | | | | |
| --- | --- | --- | --- | --- | --- | --- |
| **Recruitment and screening**  <8-24 weeks antenatal> | **Consent and baseline assessment** <12-25 weeks antenatal> | **Randomisation**  <14-27 weeks antenatal> | **Intervention or control**  <20-38 weeks antenatal> | **Intervention or control**  <6-24 weeks postnatal> | | **Follow up visit**  <26-52 weeks postnatal> |
| Review inclusion and exclusion criteria | Informed consent obtained | Participant notified of trial arm | Participants randomised to ETPB invited to attend 4 antenatal group sessions, 3 postnatal one to one sessions and a final group session | **Sub-sample of ETPB participants participate in individual semi-structured interviews and telephone interview aboutantental and home-based/ telephone postnatal sessions** | | Follow-up questionnaire completed, including primary endpoints:   - Hospital Anxiety and Depression Scale - Outwardly expressed irritability questions from Adult Wellbeing Scale |
| Participant given ‘at a glance leaflet’ | Baseline questionnaire completed | GP notified of trial arm | Participants randomised to MB invited to attend 7 antenatal group sessions and a final postnatal group session | **Sub-sample of MB participants participate in telephone interview about postnatal sessions (selected group-based sessions are observed)** | | Service use diary completed |
| Permission given to provide THRIVE team with participant contact details, CHI number and GP details |  | Referring professional notified of trial arm | **ETPB participants complete pre-intervention questionnaire, session evaluation forms and post-intervention questionnaire** |  | | Video of mother-infant play interaction recorded to assess CARE Index primary endpoint |
| Recruitment to trial information sheet posted or given to participant by research team before baseline visit and informed consent occurs. |  |  | **MB participants complete pre-intervention questionnaire, session evaluation forms and post-intervention questionnaire** |  | | Video of mother-infant care-giving episode (MPOS) |
|  |  |  | **Sub-sample of ETPB, MB and CAU participants invited to participate in semi-structured 1-2-1 interview about antenatal care, intervention attendance and parenting expectations** | **Sub-sample of ETPB, MB and CAU participants invited to participate in semi-structured 1-2-1 interview about postnatal care, intervention attendance and parenting experiences 3-12 months post-natally.** | | |
|  |  |  |  |  |  | |
|  |  |  | **Sub-sample of complete delivery of selected ETPB and MB groups observed .** |  |  | |

| **SCHEDULE OF ACTIVITIES FOR ETPB AND MB FACILITATOR S** | | | | | | | |
| --- | --- | --- | --- | --- | --- | --- | --- |
| **Recruitment and screening** | **Consent and baseline assessment** | **Allocation** | **Follow up 1** | **Intervention training** | **Follow up 2** | **Delivery and supervision** | **Follow up 3**  Experienced facilitator (3 or more groups delivered) |
| NHS Greater Glasgow and Clyde advertise for facilitator s to deliver parenting support interventions as part of THRIVE Trial | Facilitator s provided with recruitment to trial information and consent booklet and asked to provide consent for collection of research data across trial life course | Facilitator s without previous training in Triple P or Mellow Parenting interventions randomised to deliver either ETPB or MB | **Facilitator s complete pre-training questionnaire** | Facilitator s trained in delivery of either MB or ETPB | **Facilitator s complete post-training questionnaire** | Facilitator s invited to deliver around 3 intervention groups each. | **Facilitator s complete post-training questionnaire** |
| NHS Ayrshire and Arran advertise for facilitator s to deliver parenting support interventions as part of THRIVE Trial |  | Facilitator s with previous training in MB or Mellow Parenting interventions allocated to deliver MB | **Sub-sample of facilitator s invited to participate in semi-structured interview with THRIVE researcher** |  |  | **Facilitator s collect pre-intervention, session evaluation and post-evaluation questionnaires from participants attending groups** | **Sub-sample of facilitator s invited to participate in semi-structured interview with THRIVE researcher** |
|  |  | Facilitator s with previous training in Triple P for Baby or Triple P interventions allocated to deliver ETPB |  |  |  |  |  |
|  |  |  |  |  |  | **Sub-sample of facilitator s observed delivering group by THRIVE researcher** |  |
|  |  |  |  |  |  | Facilitator s attend top-up training and supervision sessions as required. | Supervisors of MB and ETPB participated in individual interviews |
